# Editorial: Community series in antimicrobial peptides: Molecular design, structure function relationship and biosynthesis optimization

**DOI:** 10.3389/fmicb.2023.1125426

**Published:** 2023-01-16

**Authors:** Na Yang, Rustam Aminov, Octavio Luiz Franco, Cesar de la Fuente-Nunez, Jianhua Wang

**Affiliations:** ^1^Innovative Team of Antimicrobial Peptides and Alternatives to Antibiotics, Feed Research Institute, Chinese Academy of Agricultural Sciences, Beijing, China; ^2^Gene Engineering Laboratory, Feed Research Institute, Chinese Academy of Agricultural Sciences, Beijing, China; ^3^Key Laboratory of Feed Biotechnology, Ministry of Agriculture and Rural Affairs, Beijing, China; ^4^The School of Medicine, Medical Sciences and Nutrition, University of Aberdeen, Aberdeen, United Kingdom; ^5^S-Inova Biotech, Universidade Católica Dom Bosco, Campo Grande, MS, Brazil; ^6^Centro de Análises Proteômicas e Bioquímicas Programa de Pós-Graduação em Ciências Genômicas e Biotecnologia, Universidade Católica de Brasília, Brasília, DF, Brazil; ^7^Machine Biology Group, Departments of Psychiatry and Microbiology, Institute for Biomedical Informatics, Institute for Translational Medicine and Therapeutics, Perelman School of Medicine, University of Pennsylvania, Philadelphia, PA, United States; ^8^Departments of Bioengineering and Chemical and Biomolecular Engineering, School of Engineering and Applied Science, University of Pennsylvania, Philadelphia, PA, United States; ^9^Penn Institute for Computational Science, University of Pennsylvania, Philadelphia, PA, United States

**Keywords:** antimicrobial peptides, mining and learning, structure function relationship, heterologous expression, druggability

The continuous rise in antimicrobial resistance during the last decades has significantly contributed to the R&D of alternatives such as antimicrobial peptides (AMPs). In the first volume of this topic, we proposed a combinatorial approach involving AMPs, antimicrobials and vaccines, which would be instrumental for the prevention and treatment of human and animal diseases within the One Health framework (Figure 1) (Wang et al., 2018, 2019; Cardoso et al., 2019a; Yang et al., 2019; Ma et al., 2021; Wu et al., 2021; Zheng et al., 2021; Hao et al., 2022). Compared to conventional antimicrobials, AMPs possess certain advantages such as high penetration and internalization, in some cases decreased likelihood of resistance emergence among bacterial pathogens, and lower probability of accumulation in tissues (Wang et al., 2019, 2022; Aminov, 2022). Selective inhibition of bacterial pathogens without causing significant cytotoxic effects, is not only an essential requirement but also a critical challenge for the R&D of AMPs. The charge, special amino acid (aa) residues, hydrophobicity/hydrophilicity ratio and secondary structure directly affect the antibacterial activity, stability and cytotoxicity of AMPs. Thus, the discovery of new AMPs by natural screening, database mining and machine learning, in addition to the rational structural design of these agents could greatly contribute to translating them from lab to clinic. These exciting new developments are highlighted in 21 papers published in the second volume of the community series of Research Topics devoted to AMPs.

**Figure 1:**
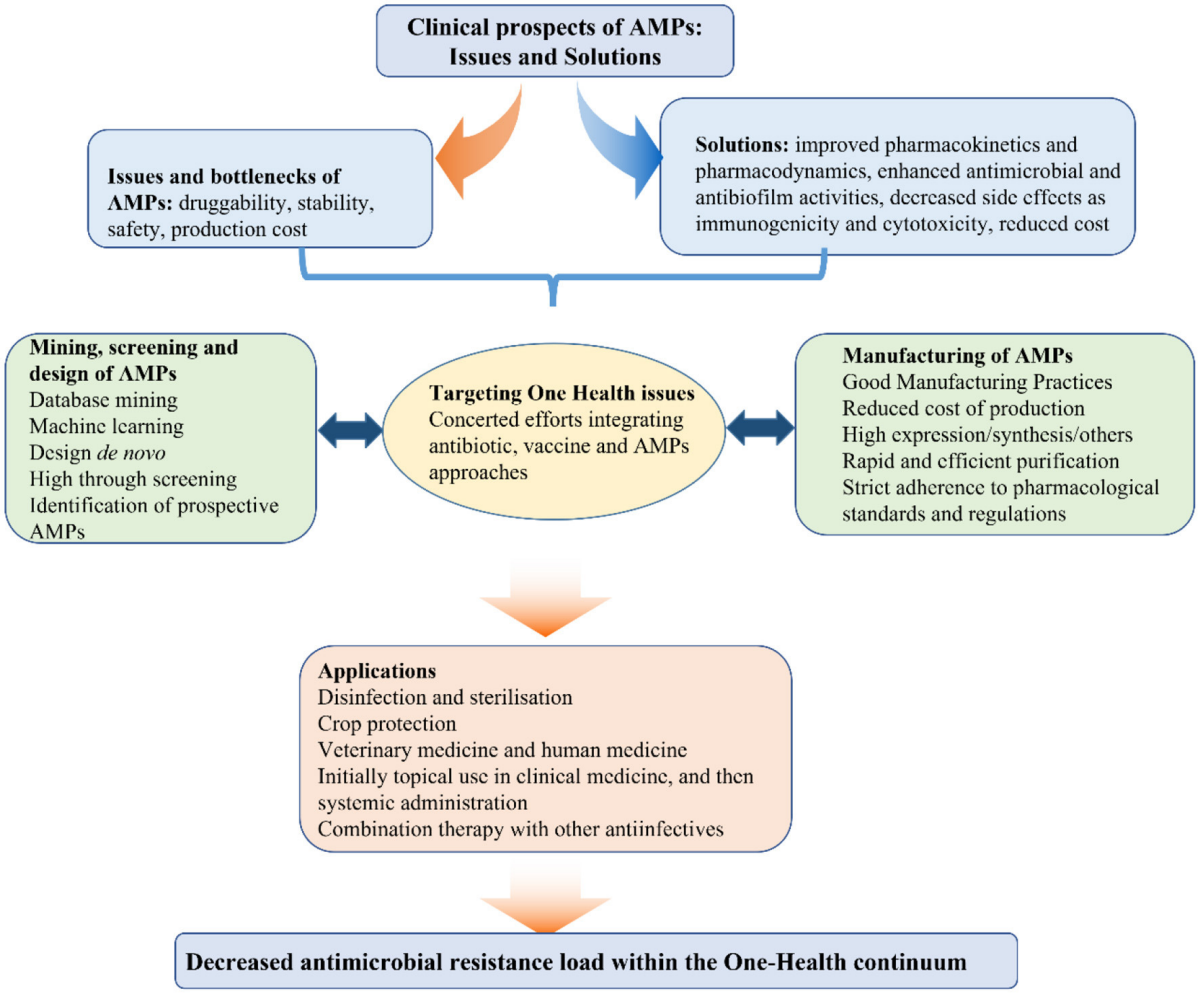
Framework of AMPs development for the post-antibiotic era.

## Discovery of natural AMPs

AMPs are short natural molecules, which are encountered in the majority of living organisms and serve as a first line of defense. According to the Antimicrobial Peptide Database (APD, https://aps.unmc.edu/), AMPs have been discovered from six life kingdoms: bacteria, archaea, protists, fungi, plants, and animals. They are mostly of animal origin, with 74% of known natural AMPs isolated from this source (Wang et al., 2016; Wang, 2022). AMPs in animals serve as host defense peptides to prevent pathogen invasion. Liu M. et al. established the coding sequences (CDS) and deduced the full-length amino acid sequences of novel hepcidin peptides from Antarctic Notothenioid Fish. The mature hepcidin peptides four disulphide bonds, differing from the typical defensins (α, β, and Θ) with three such bonds. This AMP was successfully expressed in *Escherichia coli* and displayed a broad-spectrum antibacterial activity. Microorganisms also produce AMPs to defend their ecological niches. Compared to animal AMPs, however, the biosynthetic pathways of microbial AMPs are divided into ribosomally produced and non-ribosomally produced peptides, yielding a great diversity of structural types of AMPs. Wu Y.-p. et al. isolated a cyclic non-ribosomal lipopeptide polymyxin A1 from *Paenibacillus thiaminolyticus* and determined the biosynthetic gene cluster for its synthesis, which included five open reading frames (ORFs). The lipopeptide structure confers stability and strong activity against Gram-negatove bacteria. This AMP, however, should be further tested for its efficacy and toxicity *in vivo*. Bacteriocins are ribosomally produced AMPs, which primary function is to inhibit competing strains present within the same ecological niche. Thus, they display a narrow activity range, essentially directed toward close relatives. This property could be advantageous, allowing precision therapy and infection control. Vogel et al. isolated a bacteriocin Angicin from *Streptococcus anginosus*, which is not subjected to posttranslational modifications (Vogel et al., 2021). Angicin displays no cytotoxicity toward eukaryotic cells since it precisely targets the bacterial mannose phosphotransferase system (Man-PTS), and there is no identified target in eukaryotic cells (Vogel et al.). Previous studies on AMPs have been mainly focused on their antimicrobial effect against bacteria, but some recent works have also involved fungi. Fungi are frequently plant pathogens, and there has been a considerable interest in using microorganisms or their compounds as a sustainable bio-control measure for the protection of plants against fungal diseases. Zhu H. et al. identified the antifungal tetrapeptide His-Ala-Phe-Lys (Hafk) from the bacterium *Burkholderia arboris* by using a Tn5 transposon mutation library. Inactivation or deletion of the *cobA* gene resulted in a reduced antifungal activity and significantly decreased the production of Hafk. Thus, the Hafk peptide has a significant potential as a biocontrol agent for crop fungal diseases. Presently, however, the natural reservoirs of AMPs such as the marine environment have not been sufficiently explored. The discovery and development of novel AMPs from under-explored ecological niches would certainly contribute to the health initiative within the One Health framework (Travis et al., 2018; Lazzaro et al., 2020; Hao et al., 2022).

## Structure and function of AMPs

Natural AMPs are the product of long-term evolution and they have evolved to perform certain functions such as providing protection to against infectious invasion or occupation of ecological niches. These functions are not always in-line with our needs, and here we can investigate the structure-and-function relationships of AMPs in order to improve their characteristics for our purposes. Mehamycin, a drosomycin-type antifungal peptide (DTAFP), belongs to the defensin-type family present in plants and ecdysozoans. By analyzing sequence and structural features of Mehamycin and other peptides in the DTAFP family, an 18-aa residue single Disulfide Bridge-linked Domain (sDBD) insert was identified (Gu et al.). Mutational analysis suggested a key role played by this insert in broadening the antimicrobial spectrum, accelerating pathogen eradication and thus conferring an evolutionary advantage. Identification of allosteric residues uncovered the structure-and-function trade-off. Besides the effect of peptide segments on structure and function of AMPs, single aa residues may also affect their biological function. This especially concerns aa with unique properties such as hydrophobic and basic aa. Sultana et al. investigated the role of basic aa residues, K58 and K59 and the N-terminal α-helix containing residues K7 and K30, in the antimicrobial activity of Angiogenin 4. Mutations in these positions resulted in reduced antimicrobial activity against *Salmonella* Typhimurium. Thus, the critical basic aa residues with different functionalities rather than overall electrostatic interactions play a key role in cell binding and disruption of the bacterial membrane integrity by Angiogenin 4. Optimized AMPs may be obtained by rational design by rearranging hydrophilic and hydrophobic residues, changing net charge or through conformational changes (de Moraes et al.; Wu R. et al.; Li et al.; Yuan et al.).

## Computational mining of AMPs

At present, thousands of identified AMP sequences are deposited in public AMPs databases such as antimicrobial peptides database (APD, https://aps.unmc.edu/prediction), collection of anti-microbial peptides (CAMP, http://www.camp.bicnirrh.res.in/), database of antimicrobial activity and structure of peptides (DBAASP, https://www.dbaasp.org/home), and database of antimicrobial peptides (dbAMP, http://csb.cse.yzu.edu.tw/dbAMP/, all accessed on 14 December 2022). Their structure-and-function relationships, however, are not explored to a level that would allow their further improvement and optimization (Porto et al., 2018; Torres et al., 2021; Wan et al., 2022). In the post-genomic era, the growing number of sequences deposited in databases has become a new rich resource for discovery, modification and redesign of novel AMPs (Torres et al., 2022). Tools for such analyses include Multiple Descriptor Multiple Strategy (MultiDS) screening system and multi-task learning (MTL). They are based on physicochemical and structural parameters, strategies, and algorithms for the rapid search of new candidate AMPs from genome sequences, and these systems introduce the relationship between MIC values and other parameters, providing a new perspective for improving the antibacterial activity and other key properties of AMPs (Lee et al.; Liu L. et al.). AMPs identified by genome-based screening systems were homologous to annotated and unannotated natural AMPs, and the *de novo* design methods were implemented for optimal AMP structures. Therefore, a comprehensive screening system based on bioinformatic and artificial intelligence tools enable a high-throughput prediction of novel functional AMPs with a high potential and applicability for further wet lab work (Cardoso et al., 2020; Torres et al., 2021).

## Recombinant AMPs expression

Although chemical synthesis is an important method for the preparation of short AMPs, the high manufacturing cost is a key limiting factor, particularly for peptides > 35 aa residues and with post-translational modifications (Deng et al., 2017; Cao et al., 2018; Wibowo and Zhao, 2019). Recombinant expression systems are widely used to produce various polypeptides and proteins. For example, *Bacillus* is an excellent host that can express heterologous proteins and also produce endogenous AMPs (Ren et al.). It is worth highlighting that there is currently no universal approach to express various AMPs, and the scope and applicability of each system is limited which it is based on special vector construction involving element reform and optimization, well-resistance selection for expression host suicide from AMPs, exact cleavage and secretion, and easy purification (Mao et al., 2014; Zhang et al., 2014; Teng et al., 2015; Li et al., 2017a,b, 2020; Wang et al., 2017; Cao et al., 2018; de Oliveira et al., 2020; Liu et al., 2021; Torres et al., 2021, 2022).

## Effects of AMPs on bacteria at different growth stages

In multicellular organisms AMPs are part of innate immunity and thus serve as the first line of defense against pathogens. Compared to traditional antimicrobials, AMPs are characterized by more narrow mutant selection windows and lesser chances of emergence of bacterial resistance (Rodríguez-Rojas et al., 2014, 2018; Yu et al., 2018; Liu et al., 2021; Zheng et al., 2021; Wu et al., 2022). Activities of AMPs are usually evaluated *in vitro* with exponentially growing bacteria, but under natural conditions, bacterial growth rates are much slower (Savageau, 1983; Spaulding et al., 2017). Bacteria in stationary phase, for instance, are significantly less susceptible to antimicrobials compared to exponentially growing bacteria (Gutierrez et al., 2017; Mccall et al., 2019). Using five different AMPs and three antibiotics, Rodríguez-Rojas and Roll demonstrated that AMPs possess a better bactericidal effect on non-dividing bacteria compared to antibiotics. The authors reasoned that AMPs were selected as an antimicrobial defense strategy by metazoans precisely in part due to this desirable activity against non-dividing bacteria.

## Conclusions

Pathogen resistance to antimicrobials, especially multi-drug resistance, poses a serious worldwide public health concern due to the higher morbidity and mortality rates caused by these infections. Alternatives to antimicrobials such as AMPs attract attention due to their multifactorial mechanism of action, low propensity to select for bacterial resistance, intracellular antibacterial activity, and special synergistic with conventional antimicrobials, among other advantages (Travis et al., 2018; Wang et al., 2018; Cardoso et al., 2019b; Lazzaro et al., 2020; Ageitos et al., 2022; Aminov, 2022; Hao et al., 2022; Zhu R. et al.). Thus, the discovery, modification, reformation and *de novo* design of AMPs represent an exciting approach for infection management and control. With the use of omics technologies, combined with synthetic biology approaches and gene editing and artificial intelligence tools, the increasing number of novel AMPs with high antimicrobial efficiency and low cytotoxicity can now be mined and identified for a potential use (Melo et al., 2021; Palmer et al., 2021). It must be not overlooked that AMPs, as a part of innate immunity, play a significant role in immune responses, which may occasionally be detrimental to the host. Thus, defining the antimicrobial and immune stimulation boundaries in order to limit the latter is a priority when designing new AMPs.

Currently, some AMPs are undergoing phase II-III clinical trials (Jiang et al., 2021). Most of them are used topically for wound and skin infections. The main reason for the topical use is to restrict systemic effects that could be detrimental for the host because of the impact of AMPs on the immune system. Compared to traditional antimicrobials, many AMPs derived from animals have immune functions besides their antibacterial effect (Ganz, 2003; Nesa et al., 2020). Thus, systemic application of AMPs may potentially display side effects resulting from their innate immunomodulatory properties. In order to be considered for systemic administration, AMPs should lack off-target effects, possess desirable bioavailability, stability, and half-life profiles, and optimal pharmacokinetic methods should be established (Zheng et al.). From a synthetic biology perspective, manufacturing of AMPs is not problematic since numerous toolkits are currently available (Cao et al., 2018; Hao et al., 2018). However, the choice of expression systems for AMPs should be determined based on desired properties such as the range of microorganisms targeted, the kind of application envisaged, possibility and feasibility of heterogenous expression of these peptides, and a reasonable and competitive cost of manufacturing once AMPs are ready for clinical applications (Zhang et al., 2011; Mao et al., 2014; Teng et al., 2015; Li et al., 2017b; Cao et al., 2018; de Oliveira et al., 2020; Hao et al., 2022).

## Author contributions

The first draft text of this editorial was written by NY as assistant of JW and his co-editors with their guide and direction. All authors contributed to the article and approved the submitted version.
